# The MicroRNA828/MYB12 Module Mediates Bicolor Pattern Development in Asiatic Hybrid Lily (*Lilium* spp.) Flowers

**DOI:** 10.3389/fpls.2020.590791

**Published:** 2020-10-30

**Authors:** Masumi Yamagishi, Moeko Sakai

**Affiliations:** Research Faculty of Agriculture, Graduate School of Agriculture, Hokkaido University, Sapporo, Japan

**Keywords:** anthocyanin, color pattern, bicoloration, phased-siRNA, post-transcriptional gene regulation, subgroup 6 R2R3-MYB

## Abstract

Some Asiatic hybrid lily cultivars develop bicolor tepals, which consist of anthocyanin-pigmented upper halves and un-pigmented lower halves. *MYB12*, a subgroup 6 member of R2R3-MYB that positively regulates anthocyanin biosynthesis, is downregulated in the lower halves. However, *MYB12* is usually expressed over entire tepal regions in numerous lily cultivars. Why *MYB12* of bicolor cultivars exhibits variable expression spatially in a single tepal remains unclear. Since the lily *MYB12* mRNA harbored a binding site for microRNA828 (miR828), the involvement of miR828 in variable spatial accumulation of *MYB12* transcripts was evaluated. We analyzed the cleavage of *MYB12* mRNA, mature miR828 accumulation, and *MYB12* transcript-derived siRNA generation (microRNA-seq). In the bicolor tepals, mature miR828 was more highly accumulated in the lower halves than in the upper halves, and miR828-directed cleavage of *MYB12* transcripts was observed predominantly in the lower halves. Moreover, the cleavage triggered the production of secondary siRNA from *MYB12* transcripts, and the siRNAs were accumulated predominantly in the lower halves. Consequently, miR828 suppressed *MYB12* transcript accumulation in the white region, and the miR828/MYB12 module participated in the development of bicolor patterns in lily flowers. The results present the first example of a microRNA mediating flower color patterns. Finally, we discuss the potential of miR828 creating flower color variations through suppressing the activity of subgroup 6 R2R3-MYB positive regulators in other species.

## Introduction

Anthocyanin color patterns are often observed in flowers, including spots, stripes, venation, bud-blush, and bicoloration (two colors in a single petal). The color patterns create major variations in flower appearance and often enhance the ornamental value of flowers. Consequently, the exploration of the underlying mechanisms involved in the development of anthocyanin color patterns could facilitate the breeding of floricultural crops (Nakayama, 2014). Mechanisms of development of anthocyanin color patterns have been reported in some plant species, mainly model plants (Davies et al., 2012; Glover et al., 2013; Albert et al., 2014). The primary mechanism is transcriptional regulation of anthocyanin biosynthesis genes. Subgroup 6 members of R2R3-MYB transcription factors in numerous species and subgroup 5 members of R2R3-MYB transcription factors in orchids predominantly regulate anthocyanin biosynthesis in flowers (the grouping of R2R3-MYBs is according to Stracke et al., 2001), and single plant species often have a couple of *R2R3-MYB* genes grouped into the subgroups. Each of the genes exhibits spatially and temporally distinct expression profiles and causes restricted pigment deposition, resulting in the development of varied color patterns. For example, petunias have four subgroup 6 *R2R3-MYB* genes: *PhAN2* paints the entire petal region, *PhAN4* causes pigmentation in the flower tubes, and DEEP PURPLE and PURPLE HAZE are responsible for venation and bud-blush pigmentation patterns, respectively (Albert et al., 2011). In *Phalaenopsis*, three subgroup 5 *R2R3-MYB* genes regulate full-red pigmentation, red spots, and venation patterns, respectively, in petals (Hsu et al., 2015).

Lilies (*Lilium* spp.) are among the most commercially valuable and globally cultivated floriculture crops (Ministry of Agriculture, Forestry and Fisheries of Japan, 2019). Interspecific hybridization is the principal method of lily breeding. The *Lilium* species are classified into several sections, and Asiatic hybrid lilies (*Lilium* spp.) are derived from crosses among species in Sinomartagon and Daurolirion section (Yamagishi and Nakatsuka, 2017; Marasek-Ciolakowska et al., 2018). Since lilies are a major floricultural crop, genetic evaluation of their agricultural traits facilitates the improvement of the character traits of cultivars. However, lilies are not model species, and their genetic evaluation is challenging considering *Lilium* species have a large genome size (33–36 Gb; Bennett and Leitch, 2011) and long life cycles (3–7 years from sowing to anthesis), and its stable transformation remains challenging.

A high variation in flower color hues is the major selling point for Asiatic hybrid lilies, which accumulate anthocyanins (cyanidin 3-rutinoside, Nørbæk and Kondo, 1999) and carotenoids (Wang and Yamagishi, 2019) in flowers. In addition, a variety of anthocyanin color patterns, such as restricted pigmentation at tepal bases (large spots or blotches) or the upper halves (bicolor), and several types of spots, including raised spots, splatter-type spots, and brush mark spots, are often observed in Asiatic hybrid lily cultivars (Yamagishi, 2013). The underlying mechanisms of such color patterns have been evaluated. Several subgroup 6 members of the *R2R3-MYB* genes are expressed in lily flowers: *MYB12* usually paints entire tepal regions (Yamagishi et al., 2010), *MYBSPLATTER* (formerly *LhMYB12-Latvia*, Yamagishi, 2020a) causes splatter-type anthocyanin spots (Yamagishi et al., 2014), *MYB19Short* and *MYB19Long* are responsible for raised-spot pigmentation (Yamagishi, 2020b), *MYB19Long* is indispensable for brush mark pigmentation (Yamagishi, 2020c), and *MYB18* is involved in the development of large spots (blotches) at tepal bases (Yamagishi, 2018). Therefore, transcriptional regulation, governed by subgroup 6 members of *R2R3-MYB* genes, is the primary mechanism of the establishment of a variety of color patterns in lilies.

**Figure 1:**
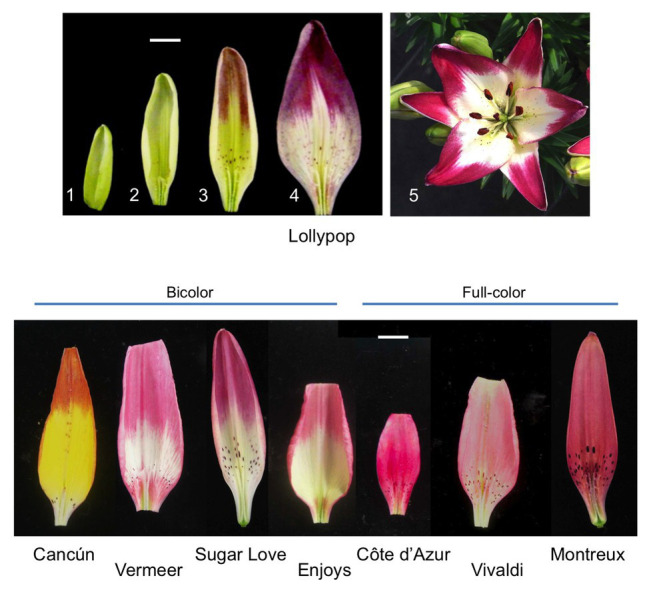
**Upper panels**: Inner tepals (adaxial surface) of the Lollypop cultivar at floral development stages 1, 2, 3, and 4 and a whole flower of Lollypop at anthesis (stage 5). **Lower panel**: Inner tepals (adaxial surface) of four bicolor (Cancún, Vermeer, Sugar Love, and Enjoys) and three full-color (Côte d’Azur, Vivaldi, and Montreux) Asiatic hybrid lily cultivars at flower bud development stage 4. The yellow color of Cancún tepals is derived from carotenoids. White bar = 1 cm.

Some Asiatic hybrid lily cultivars, including Lollypop, develop bicolor tepals where anthocyanin accumulates in the upper halves of tepals but scarcely accumulates at the lower halves of tepals (Figure 1). We have previously compared transcriptome profiles between the upper and lower halves of Lollypop tepals and clarified that the entire anthocyanin biosynthesis pathway is suppressed at the lower halves (Suzuki et al., 2016). Therefore, the bicolor pattern in Lollypop is caused by transcriptional regulation of anthocyanin biosynthesis genes. Putative transcription factors that positively or negatively regulate anthocyanin biosynthesis are surveyed using transcriptome data, among which, certain negatively regulating transcription factors are expressed in Lollypop tepals, including R3-MYB suppressors (Sakai et al., 2019). If such negative regulators are involved in bicolor formation, their expression would be expected to be high in white tepal regions. To date, however, there have been no reports of putative suppressors exhibiting higher expression in the unpigmented regions of flowers (Suzuki et al., 2016; Sakai et al., 2019). Three R2R3-MYB sequences belonging to subgroup 6 are included in the Lollypop transcriptome. Among the three R2R3-MYB sequences, *MYB12* is expressed mainly in the tepals, and its expression levels are higher in the upper halves than in the lower halves of the tepals, suggesting that MYB12 is the main transcription factor causing varying anthocyanin biosynthesis activities, and mediating bicolor anthocyanin pigmentation patterns (Suzuki et al., 2016). However, since *MYB12* is often responsible for the pigmentation of whole tepal regions in many lilies (Yamagishi et al., 2010, 2012; Yamagishi, 2011), it remains unclear why and how *MYB12* expression is suppressed in the lower halves of bicolor tepals.

MicroRNAs are non-coding RNA species that play key roles in plant development and stress responses. MicroRNAs usually suppress the activity of target genes post-transcriptionally through cleavage of the transcripts or translational attenuation. Many transcription factor genes, including MYB family genes, are targets of microRNAs. MicroRNA production starts from primary microRNA (pri-miRNA), which is cleaved in the nucleus into smaller precursor microRNA (pre-miRNA) with a hairpin structure. These pre-miRNAs are then exported to the cytoplasm and cleaved by DICER-LIKE 1 (DCL1) to form 20–24 nucleotide-long mature microRNAs. Subsequently, the mature microRNAs are loaded into the ARGONAUTE (AGO) effector protein, a part of RNA-induced silencing complexes (RISCs). Afterward, the mature microRNAs guide the RISCs to target mRNA(s) through sequence complementarity and eventually mediate gene silencing (Zhang, 2015; D’Ario et al., 2017). In addition, some 22 nucleotides-long microRNAs are capable of initiating secondary small interfering RNAs (siRNAs). The RNA fragments downstream (or upstream in rare cases) of the cleaved site are converted into a double-stranded RNA molecule by RNA-dependent RNA polymerase 6, which is processed by the DCL4 enzyme into approximately 21 nucleotide-long siRNAs, which are called phased siRNAs (phasiRNAs). Some phasiRNAs can further regulate the expression of other target genes in *trans* and are called *trans*-acting siRNAs (tasiRNAs; Shivaprasad et al., 2012; Fei et al., 2013; Deng et al., 2018).

Among the microRNAs identified in a wide range of plant species, miR828, miR858, and miR159 are capable of targeting *R2R3-MYB* genes (Li and Lu, 2014; Jia et al., 2015; Sharma et al., 2016). Among them, miR828 often suppresses the expression of *R2R3-MYB* genes belonging to subgroup 15 and subgroup 4, which negatively regulate anthocyanin accumulation (Xia et al., 2012; Zhu et al., 2012). Therefore, anthocyanin pigment amounts increase in fruits or tubers with an increase in miR828 accumulation (Bonar et al., 2018; Tirumalai et al., 2019). However, in *Arabidopsis*, miR828 directly targets *AtMYB113* and indirectly targets *AtMYB75* (*AtPAP1*) and *AtMYB113* through TAS4-siR81(-), one of the tasiRNAs derived from Trans-Acting SiRNA Gene 4 (*TAS4*) transcripts (Rajagopalan et al., 2006). Since *AtMYB75* and *AtMYB113* are subgroup 6 members of R2R3-MYB that positively regulate anthocyanin biosynthesis, miR828 and TAS4 suppress anthocyanin biosynthesis and are involved in feedback regulation of anthocyanin accumulation in vegetative tissues under stress conditions (Luo et al., 2012).

An miR828 target sequence was found in the nucleotide sequence of lily *MYB12* (shown in the results section); however, sequences potentially recognized by miR159, miR858, or TAS4-siR81(-) were not detected. In addition, partial sequences of *pri-MIR828* were included in the Lollypop transcriptome (Suzuki et al., 2016). Therefore, the potential of miR828 to suppress *MYB12* expression predominantly in the white tepal region was investigated in the present study. We demonstrated that lily miR828 was highly accumulated in the white tepal region and cleaved *MYB12* mRNA. *MYB12* transcript-derived secondary siRNA molecules investigated using microRNA-seq were also highly accumulated in the white region. Consequently, we conclude that the miR828/MYB12 module has a key role in the development of bicolor tepals in lilies.

## Materials and Methods

### Plant Materials

Five Asiatic hybrid lily bicolor cultivars, Lollypop, Cancún, Vermeer, Sugar Love, and Enjoys, and three full-color cultivars, Côte d’Azur, Vivaldi, and Montreux (2*n* = 2x = 24), were examined in the present study (Figure 1). The lily plants were grown in a greenhouse (unheated and natural photoperiod) in the experimental farm of Hokkaido University, Sapporo, Japan. The flower bud developmental stages were defined according to Nakatsuka et al. (2003), i.e., no obvious anthocyanin pigmentation in stage 1, emergence of anthocyanin color only on raised spots in stage 2, beginning of anthocyanin coloration of adaxial tepal surface in stage 3, full pigmentation of adaxial tepal surface in stage 4 (1 day before anthesis), and flower blossoming in stage 5 (Figure 1). Tobacco plants (*Nicotiana tabacum* “Xanthi NC”) were grown in a growth chamber at 20°C under a 16-h light-8-h dark photoperiod.

### Extraction of RNA

Low molecular weight (LMW) RNA was isolated from lily organs using a High Pure miRNA Isolation Kit (Roche Diagnostics K. K., Tokyo, Japan). Total RNA was extracted from lily tepals and tobacco leaves using a NucleoSpin® RNA kit (MACHEREY-NAGEL GmbH & Co. KG, Düren, Germany).

### Sequencing

The full-length cDNA sequence of *pri-MIR828* was obtained by rapid amplification of cDNA ends (RACE)-PCR (Lai et al., 2012) using gene-specific primers (Supplementary Table S1) designed using the partial *pri-MIR828* sequence (Suzuki et al., 2016) and total RNA isolated from Lollypop tepals. The amplified fragments were cloned into the pGEM-T Easy Vector (Promega, Tokyo, Japan) and sequenced [DNA DataBank of Japan (DDBJ) accession numbers LC569960 and LC569961]. The nucleotide sequences were aligned using the default parameters in Clustal W in the Kyoto University Bioinformatics Center website.^1^

RNA ligase mediated (RLM)-RACE PCR was carried out to validate the targets of miR828. RNA adapter and total RNA isolated from lily tepals or tobacco leaves were ligated using RNA ligase (Takara, Otsu, Japan). The ligated RNA was used for cDNA synthesis using the reverse primer of *MYB12* and PrimeScript II reverse transcriptase (Takara). Nested PCR was performed using the two sets of primers. Sequences of the RNA adapter and primers are listed in Supplementary Table S1. The amplified fragments of the appropriate size were cloned into the pMD20-T vector (Takara) and sequenced.

### *Agrobacterium*-Mediated Transient Assay (Agroinfiltration)

The putative *pre-MIR828* sequence (141 bp), which included the guide and passenger strands, and made a hairpin structure, was PCR-amplified using primers containing *Xba*I or *Sac*I recognition sites (Supplementary Table S1). Subsequently, the *MIR828* fragment was inserted downstream of the constitutive cauliflower mosaic virus 35S promoter (35S-p) using the restriction sites. Other plasmid constructs were derived from Yamagishi (2016, 2018).

*Agrobacterium tumefaciens* (EHA105) harboring the 35S-p::*MIR828* construct and harboring the 35S-p::*MYB12* construct were separately or simultaneously infiltrated into tobacco leaves using a syringe (Suzuki et al., 2015). In another experiment, *A. tumefaciens* harboring 35S-p::*MIR828*, 35S-p::*MYB12*, 35S-p::*LhbHLH2*, or *Lilium* hybrid dihydroflavonol 4-reductase (*LhDFR*)-promoter::intron-containing ß-glucuronidase (*iGUS*) constructs were infiltrated into several combinations. Six or three days after infiltration, leaf segments were harvested and gene expression levels were evaluated by quantitative reverse transcription PCR (qRT-PCR).

### Expression Analysis

Mature miR828 was transcribed into cDNA by reverse transcribing LMW RNA using the stem-loop pulsed RT protocol and, then, end-point PCR was performed to amplify 60-nt fragments that included the mature miR828 sequences (Varkonyi-Gasic et al., 2007) using primers listed in Supplementary Table S1. To confirm the quality of LMW RNA, we amplified *U6* small nuclear RNA by reverse transcribing LMW RNA with the U6br primer, followed by PCR amplification with the primers U6af and U6br (Supplementary Table S1). For the analysis of mRNA accumulation, cDNA was synthesized from total RNA using the ReverTraAce® qPCR RT Master Mix with gDNA Remover (Toyobo, Tokyo, Japan).

Quantitative reverse transcription PCR of mature miR828, *pri-MIR828*, anthocyanin biosynthesis genes [chalcone synthase a (*CHSa*), *CHSb*, flavanone 3-hydroxylase (*F3H*), *DFR*, and anthocyanidin synthase (*ANS*)], and other protein-coding sequences were conducted using the THUNDERBIRD® SYBR® qPCR Mix (Toyobo) and PCR primers listed in Supplementary Table S1. Signals were monitored using the CFX Connect Real-Time System (Bio-Rad, Tokyo, Japan). The amount of lily *U6*, lily *ACTIN*, tobacco ubiquitin (*UBQ*), or *Renilla* luciferase (*Rluc*) on the LhDFR-promoter::*iGUS* construct (agroinfiltration) in each sample was used to normalize the amount of each target mRNA, using the formula 2^−∆Ct^, where ∆Ct = Ct(target gene) − Ct(reference gene). Five or three biological replicates were used to calculate the mean values and standard error. The statistical differences were analyzed using Tukey’s honestly significant difference (Tukey’s HSD) test using R v3.3.1^2^ or the *t*-test.

### Small RNA Sequencing

Low molecular weight RNA isolated from the lower and upper halves of Lollypop tepals at stage 4 was processed into sequencing libraries using adapted Illumina protocols and sequenced by Hokkaido System Science Co., Ltd. (Sapporo, Japan) using an Illumina HiSeq sequencer (Illumina Inc., CA, USA). FASTQ file generation, adapter removal, and paired-read merging were performed using Cutadapt (Martin, 2011) and Fastq-join.^3^ The obtained sequences were plotted (allowing 100% homologous matching) onto the sense and antisense orientations of the *MYB12* mRNA sequence using Bowtie (Langmead et al., 2009).

## Results

### Feature of Lily MicroRNA828

Lily miR828 was characterized in the present study to investigate the underlying mechanism of suppression of the expression of *MYB12* at the lower halves of Lollypop tepals. First, the primary transcripts of *MIR828* were defined using 5'- and 3'-RACE PCR. Two sequences (636 and 696 bp) were detected: guide and passenger strands appeared at the 5'-terminal region with identical sequences. However, sequences downstream of the region were slightly different (Supplementary Figure S1). As lily cultivars are genetically heterozygous, the two sequences should be allelic. RNA structure^4^ predicted that *pre-MIR828* formed a hairpin loop structure. The two sequences exhibited similar structures (the structure of *pri-MIR828-2* is presented in Supplementary Figure S2). One mismatched base pair and one bulged base were observed in the duplex of guide and passenger strands.

Target genes of miR828 in lily were predicted at the “psRNATarget: A Plant Small RNA Target Analysis Server” website^5^ (Dai et al., 2018), mainly using the Lollypop tepal transcriptome data as a target file. Fifteen sequences were detected under an expectation value lower than 4 (Supplementary Table S2). Out of the 45 *R2R3-MYB* genes expressed in Lollypop tepals (Suzuki et al., 2016; Yamagishi, 2020b), five were predicted as potential targets, including the all subgroup 6 members (*MYB12*, *MYB15like*, *MYB16*, and *MYB19S*).

### Lily MicroRNA828 Cleaves *MYB12* mRNA in Tobacco Leaves

**Figure 2:**
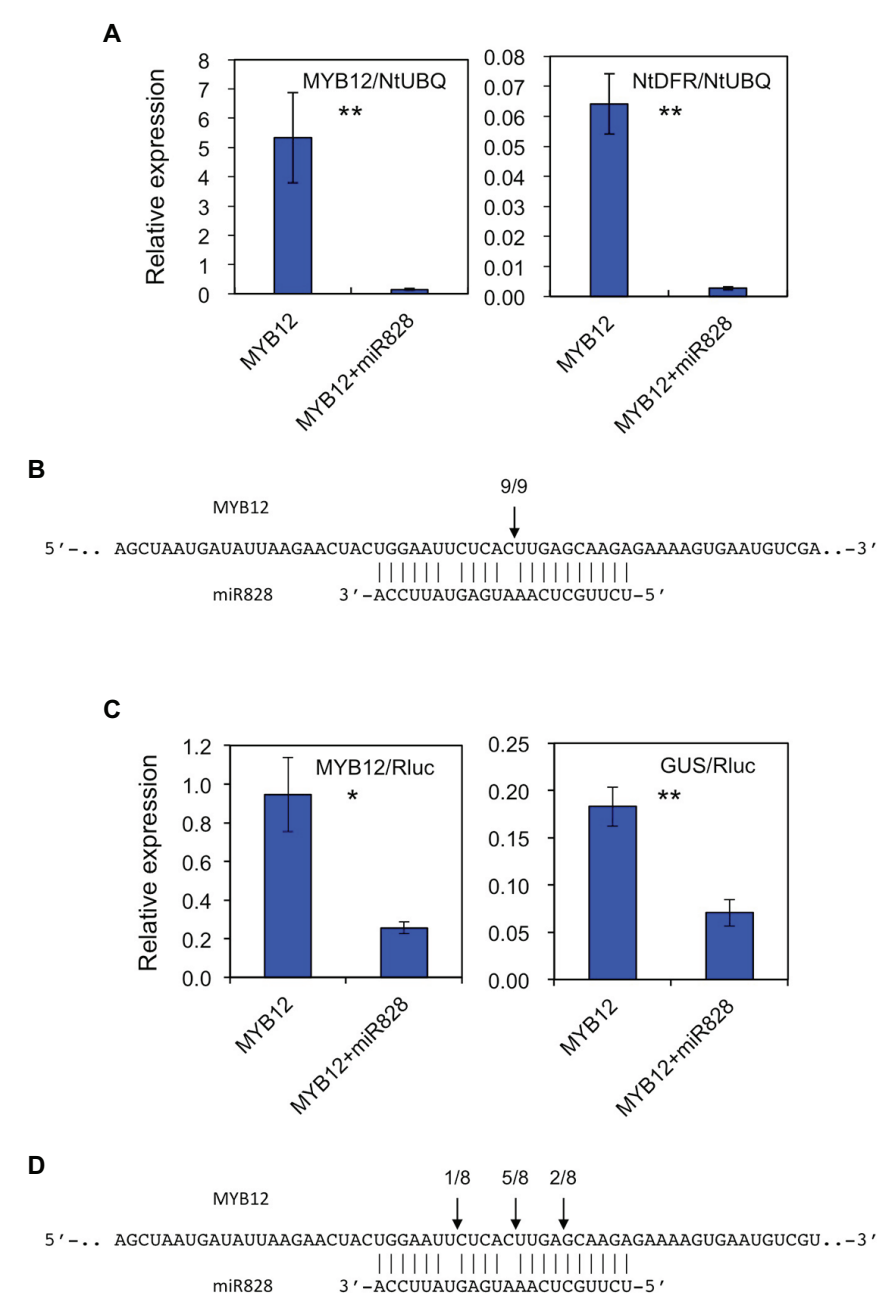
**(A)** Relative expression levels of *MYB12* and *NtDFR* in tobacco (*Nicotiana tabacum*) leaves (five plants) infiltrated with *Agrobacterium* harboring 35S-p::*MYB12* or *Agrobacterium* with 35S-p::*pre-MIR828* plus *Agrobacterium* carrying 35S-p::*MYB12*. The levels of expression were analyzed 6 days after infiltration. The tobacco UBIQUTIN (*NtUBQ*) gene was used to normalize the expression of target genes. **(B)** The cleavage site (arrow) in *MYB12* mRNA predicted using RNA ligase mediated (RLM)-rapid amplification of cDNA ends (RACE) PCR (RLM-RACE PCR). **(C)** Relative expression levels of *MYB12* and *GUS* in tobacco leaves (three plants). A construct harboring 35S-p::*MYB12* or a construct containing 35S-p::*pre-MIR828* plus a construct containing 35S-p::*MYB12* were introduced in tobacco leaves using *Agrobacterium*, together with 35S-p::*LhbHLH2* and lily DFR-p::*iGUS* constructs. Expression levels were examined 3 days after infiltration. The NOS-p-driven *Rluc* gene in the DFR-p::*iGUS* construct was used to normalize the expression levels of target genes. **(D)** The cleavage sites (arrows) in *MYB12* mRNA predicted by RLM-RACE PCR. In **(A,C)** the vertical bars indicate the standard errors of the means of five or three plants. ^*^ and ^**^ Indicate significant differences at the 5 and 1% significance levels, respectively (*t*-test). In **(B,D)** segments of the *MYB12* (upper) and mature miR828 (lower) sequences are shown. Numerals at the arrows indicate the number of reads with 5' terminal ends at arrowed sites/total number of reads.

The putative *pre-MIR828* sequence was cloned into a pBI121 vector under the control of the 35S promoter to perform transient expression assays in *N. tabacum* to verify the biological activity of Lily miR828. To that end, a construct harboring 35S-p::*MYB12* was infiltrated alone or together with the 35S-p::*MIR828* construct, and the transcript abundance of *MYB12* and *NtDFR* was monitored 6 days after infiltration (Figure 2A). *NtDFR* is a tobacco endogenous gene, whose expression is stimulated by lily *MYB12* (Yamagishi, 2020a). Co-infiltration of the 35S-p::*MIR828* construct reduced the expression levels of *MYB12* and, in turn, suppressed *NtDFR* expression. RLM-RACE PCR revealed that *MYB12* mRNA was cleaved in tobacco leaves at the nucleotide corresponding to the site between the 10th and 11th nucleotides of miR828 (Figure 2B and Supplementary Figure S3A).

Similarly, the constructs harboring 35S-p::*MYB12*, 35S-p::*bHLH2* or lily *DFR* promoter-driven *iGUS* were infiltrated into tobacco leaves with or without the 35S-p::*MIR828* construct (Figure 2C). Three days after infiltration, while expression of *MYB12* activated the lily DFR promoter in tobacco leaves, co-infiltration of the 35S-p::*MIR828* construct downregulated *MYB12* expression and suppressed the promoter activity of lily *DFR*. The main cleavage site in *MYB12* mRNA was between the 10th and 11th nucleotide from the 5' end of miR828 (Figure 2D and Supplementary Figure S3B). The results illustrated in Figure 2 suggest that *MYB12* mRNA is the target of miR828 and is cleaved in the presence of mature miR828, which was processed from *pre-MIR828* in tobacco.

### MicroRNA828 Predominantly Accumulates in the White Tepal Region of Lilies

**Figure 3:**
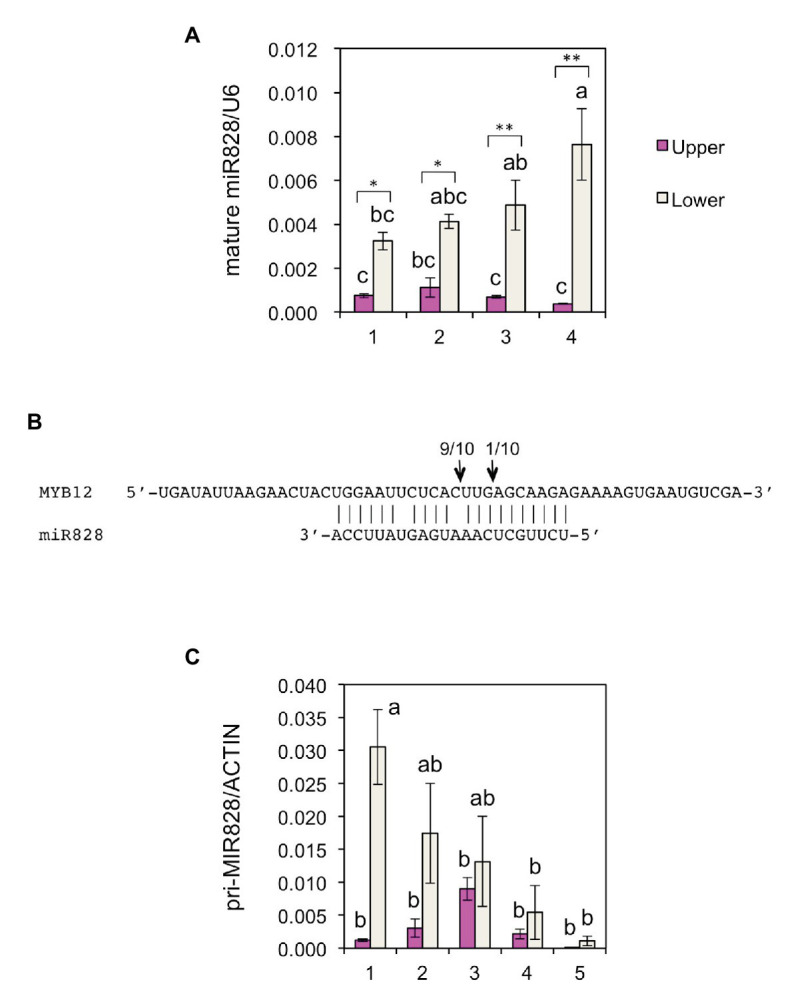
**(A)** Accumulation of miR828 [stem-loop quantitative reverse transcription PCR (qRT-PCR)] in upper and lower halves of Lollypop tepals at flower bud developmental stages 1, 2, 3, and 4. *U6* was used to normalize the accumulation. Vertical bars show the standard errors (SE) of the means of three replicates. The same letters above the columns indicate that the values are not statistically significant (*p* < 0.05) based on Tukey’s honest significant difference (HSD) test. ^*^ and ^**^ Indicate significant difference at 5 and 1% significance levels, respectively (*t*-test), between the upper and lower halves in each stage. **(B)** MiR828 cleavage site (arrows) in *MYB12*, estimated by RLM-RACE PCR. Numerals at the arrows indicate the number of reads with 5' terminal ends at arrowed sites/total number of reads. **(C)** Expression of *pri-MIR828* in upper and lower halves of Lollypop tepals at flower bud developmental stages 1, 2, 3, 4, and 5. Lily ACTIN was used to normalize the expression of the genes under identical conditions. Vertical bars show the SE of the means of three flowers. The same letters above the columns indicate that the values are not statistically significant (*p* < 0.05) based on Tukey’s HSD.

**Figure 4:**
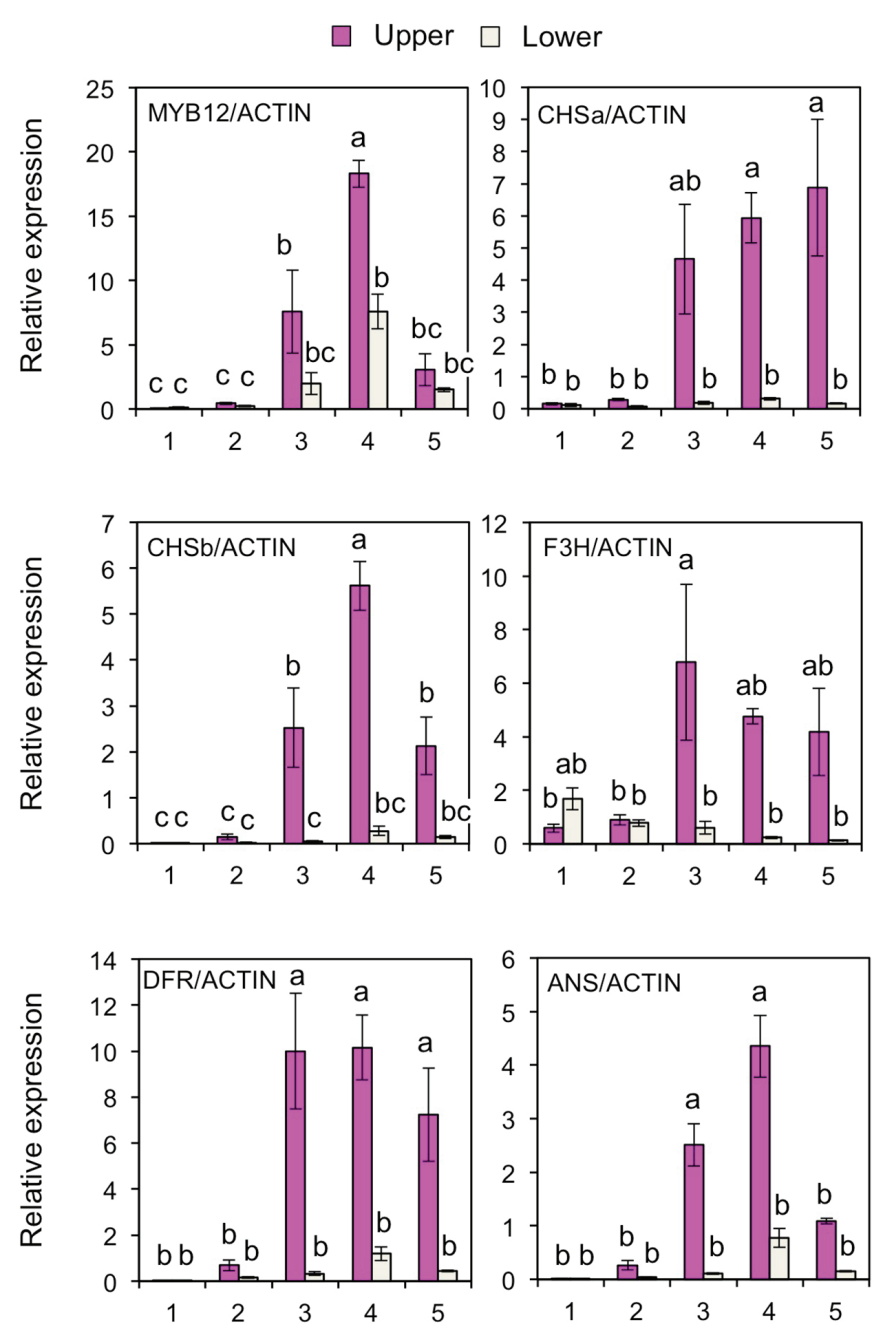
Expression of *MYB12*, *CHSa*, *CHSb*, *F3H*, *DFR*, and *ANS* genes (qRT-PCR) in the upper and lower halves of Lollypop tepals at flower bud development stages 1, 2, 3, 4, and 5. Lily *ACTIN* was used to normalize the expression of genes under identical conditions. Vertical bars represent the standard errors of the means of three flowers. The same letters above columns indicate that the values are not significantly different (*p* < 0.05) based on Tukey’s HSD test.

To further verify the post-transcriptional regulation of *MYB12*, the accumulation of mature miR828 was evaluated in lily tepals using stem-loop qRT-PCR (Figure 3A). During flower bud developmental stages from 1 to 4, its accumulation level in the lower halves increased with bud growth, while that in the upper halves was low and constant. In each of the developmental stages, accumulation levels were significantly higher in the lower halves than in the upper halves at 1 or 5% levels (*t*-test). The expression of *MYB12* and five anthocyanin biosynthesis gene was mainly detected at stages 3, 4, and 5, and, in contrast to the miR828 accumulation levels, their expression levels were higher in the upper halves than in the lower halves (Figure 4). Sequence-specific cleavage of target RNA provides strong evidence of microRNA involvement in post-transcriptional gene regulation. RLM-RACE PCR revealed that *MYB12* mRNA was cleaved in lily tepals predominantly between nucleotides that were annealed to the 10th and 11th nucleotides of miR828 (Figure 3B and Supplementary Figure S3C). Therefore, miR828 potentially contributes to the repression of *MYB12* transcript accumulation during flower bud development, resulting in the suppression of anthocyanin biosynthesis in the lower halves of tepals. *Pri-MIR828* transcript levels were estimated by qRT-PCR using primers designed at the 3' terminal region. *Pri-MIR828* expression levels were higher in the lower halves than in the upper halves and, in contrast to in mature miR828, decreased with increased flower bud development in the lower halves (Figure 3C).

**Figure 5:**
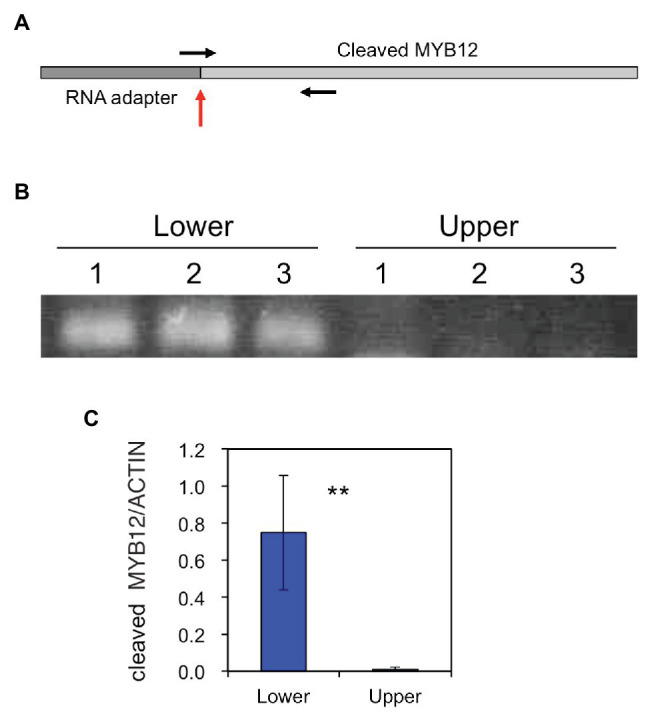
**(A)** A schematic representation of the miR828-cleaved *MYB12* fragment. The vertical red arrow indicates a putative cleavage site and horizontal arrows denote primer positions. **(B)** PCR products of miR828-cleaved *MYB12* fragments that accumulated in the lower and upper halves of Lollypop tepals. 1, 2, and 3 indicate individual flowers collected at stage 4. **(C)** Relative levels of accumulated miR828-cleaved *MYB12* estimated using qRT-PCR. The lily *ACTIN* gene was used as a reference gene. Vertical bars represent the standard errors of the means of three flowers. ^**^Indicates a significant difference at the 1% significance level (*t*-test).

The amounts of the *MYB12* mRNA-cleavage products were estimated using PCR: similar to the method of RLM-RACE PCR, RNA adapter was ligated to uncapped RNA and, after cDNA synthesis, PCR was carried out using a forward primer spanning the adapter and *MYB12* sequences and an *MYB12* gene-specific reverse primer (Figure 5). The cleaved *MYB12* transcripts were detected at relatively high levels in the lower halves of tepals but scarcely in the upper halves of tepals, indicating that MYB12 transcripts are predominantly cleaved in the white tepal region.

**Figure 6:**
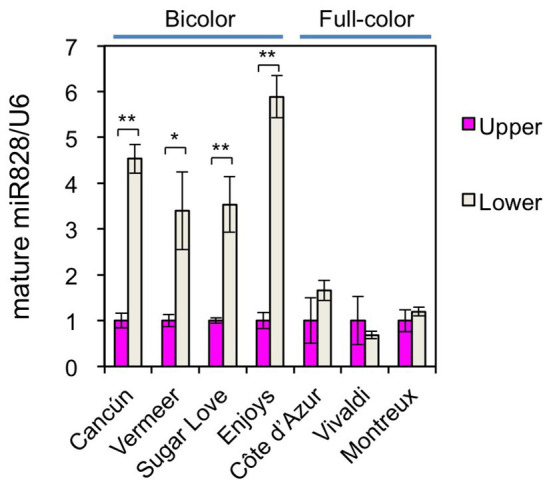
Accumulation of miR828 (stem-loop qRT-PCR) in upper and lower halves of tepals in four bicolor (Cancún, Vermeer, Sugar Love, and Enjoys) and three full-color (Côte d’Azur, Vivaldi, and Montreux) cultivars at flower bud developmental stage 4. *U6* was used to normalize the accumulation. Vertical bars represent the standard errors of the means of three replicates. ^*^ and ^**^Indicate significant difference at 5 and 1% significance levels, respectively (*t*-test), between the upper and lower halves.

To further confirm the functions of miR828 in color pattern development, the accumulation profile of miR828 was compared between bicolor cultivars and full-color cultivars (Figures 1, 6). Its accumulation levels were higher in the lower halves than in the upper halves of tepals in the four bicolor cultivars, while no clear difference was observed between the lower and upper halves of the three full-pink cultivars. Therefore, differences in miR828 levels between the two tepal sections were correlated highly with bicolor development in lily tepals.

**Figure 7:**
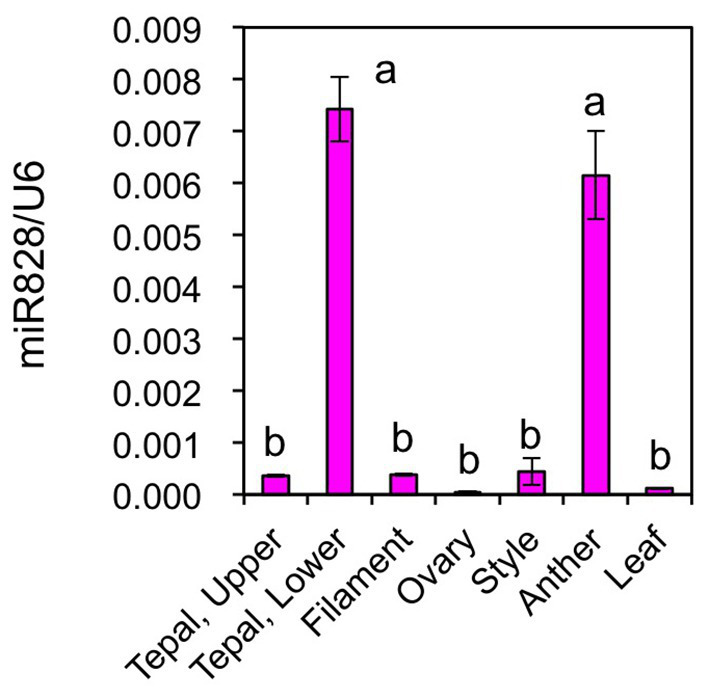
Accumulation of miR828 (stem-loop qRT-PCR) in tepals (upper and lower), filaments, ovaries, styles, anthers, and leaves of the Lollypop cultivar. Floral organs were collected at flower bud development stage 4. The *U6* gene was used to normalize accumulation levels. Vertical bars represent the standard errors of the means of three replicates. The same letters above columns indicate that the values are not significantly different (*p* < 0.05) based on Tukey’s HSD test.

Mature miR828 accumulation was compared in several Lollypop organs (Figure 7). Floral organs were collected at flower bud developmental stage 4. Its accumulation levels in filaments, ovaries, styles, and leaves were similar to those in the upper halves of tepals, while those in anthers were high, similar to those in the lower halves of tepals. Lilies accumulate high amounts of anthocyanins in anthers (Suzuki et al., 2015). In anthers, anthocyanin biosynthesis gene expression is high during the early stages of flower bud development and low at flower bud developmental stage 4. However, as *MYB12* is not expressed (Lai et al., 2012) and other positive regulators are yet to be evaluated in anthers, the significance of a high accumulation of miR828 remains unclear.

### Secondary SiRNAs Initiated by MicroRNA828-Directed Cleavage of *MYB12* mRNA

**Figure 8:**
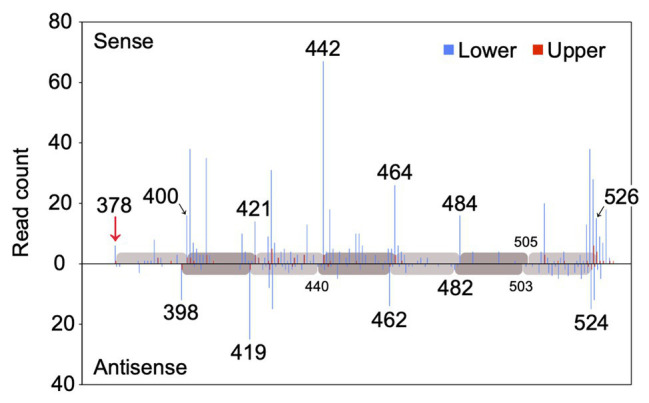
Read count of small RNA (19–24 nucleotides) mapped to the *MYB12* mRNA. The number of sequenced small RNAs with 5' residues at each position along the *MYB12* mRNA is plotted for the sense and antisense strands. Blue and red bars indicate the read counts of small RNA derived from lower and upper halves of Lollypop tepals (flower bud developmental stage 4), respectively. Numerals in the panel show nucleotide position downstream of the translation initiation site. Only the positions from the 370 to 530 nucleotides are shown. A red arrow indicates miR828-directed cleavage site. Gray and light gray bars represent 20, 21, or 22 nucleotide-intervals.

MiR828 was 22 nucleotides long. Since 22 nucleotide-long microRNAs are capable of secondary siRNA generation at their target loci (Shivaprasad et al., 2012; Källman et al., 2013), whether miR828 generated secondary siRNAs from the *MYB12* sequence was evaluated using next-generation sequencing. Small RNA was prepared from the lower and upper halves of Lollypop tepals and sequenced using Illumina technology to yield a total of 18,887,232 and 18,997,636 small RNA reads, respectively (Supplementary Table S3). Afterward, the 19–24 nucleotide-long reads were mapped to the *MYB12* sequence (Figure 8). Most of the small RNAs with the sense sequences of *MYB12* were mapped on the 3' end of *MYB12*, beginning with the miR828 cleavage site and proceeding in 20–22-nucleotide intervals. The small RNA with the antisense sequence of MYB12 was mapped in 20–22-nucleotide intervals, accounting for the 2-nucleotide offset between the sense and antisense strands. The spacing pattern is consistent with evidence that DCL4 cleaves double-stranded RNA sequentially from the end of the molecule (Yoshikawa et al., 2005) and suggests that miR828-cleavage triggers the production of secondary siRNAs from 3' cleaved transcripts of *MYB12*. MiR828-mediated phasiRNA production from *TAS4* and *R2R3-MYB* genes has been reported in *Arabidopsis*, apple, and peach (Rajagopalan et al., 2006; Xia et al., 2012; Zhu et al., 2012). Our results confirmed miR828-mediated secondary siRNA generation in monocots.

Out of each of the approximately 19 × 10^6^ small RNA reads, 874 were mapped in the lower halves and 75 in the upper halves (Figure 8 and Supplementary Table S3). The number of the mapped small RNA was more than 10-fold higher in the lower halves than in the upper halves. *MYB12* cleavage and subsequent phasiRNA production potentially occurred more frequently in the lower sections of tepals.

## Discussion

Bicolor petal patterns, which consist of pigmented and unpigmented regions in a single petal, are often observed in higher plants. Several mechanisms that create bicolor patterns have been elucidated in some species, mainly petunias. One is a post-transcriptional silencing of *CHS* genes examined in petunias (Koseki et al., 2005) and dahlias (Ohno et al., 2011). In star and picotee petunia lines, siRNA suppresses *CHS* mRNA accumulation post-transcriptionally in white petal regions, and tandemly arranged *CHS* sequences are involved in siRNA generation (Morita et al., 2012). This floral color pattern is further modified by the activation of an endogenous virus. Petunia vein clearing caulimovirus (PVCV) proviruses are activated as host plants age, and *CHS* gene silencing is suppressed by an RNA silencing suppressor of activated PVCV, resulting in anthocyanin biosynthesis in the white regions of star-type petunia petals (Kuriyama et al., 2020). Another mechanism is s space-specific flavonol synthase (*FLS*) expression. In morn petunia lines, the central regions of flowers are colorless. *FLS* levels are high, and flavonols accumulate at much higher levels than anthocyanins in the central region (Saito et al., 2006). In Lollypop tepals, the entire anthocyanin biosynthesis pathway was suppressed in the white region, and the key factor that caused the suppression was the MYB12 transcription factor. In addition, most Asiatic hybrid lily cultivars do not accumulate flavonols and flavones in tepals (Lai et al., 2012), and *FLS* was scarcely expressed in Lollypop tepals (Suzuki et al., 2016). Therefore, in the present study, we explored additional mechanisms.

Lily *MYB12* is a target of miR828 because miR828 cleaves *MYB12* mRNA in lily tepals, and *MIR828* introduced into tobacco leaves cleaved *MYB12* mRNA and suppressed *MYB12* activity. In addition, mature miR828 and the *MYB12*-derived secondary siRNA molecules accumulated more abundantly in the white region than in the pigmented region. Therefore, *MYB12* expression was significantly lower in the white tepal regions due to targeting by miR828 and potentially by the secondary siRNAs. Other microRNAs that potentially hybridized into *MYB12* mRNA were surveyed using the microRNA-seq data, but such a candidate was not found among the small RNAs of 20–24 nucleotides (data not shown). Therefore, miR828 is potentially a solo microRNA suppressing *MYB12* expression, and the miR828/MYB12 module is potentially the key mechanism causing discoloration in lily flowers.

Why does mature miR828 predominantly accumulate in the lower halves of tepals? The amounts of *pri-MIR828* transcripts were high in the lower halves and, therefore, spatially variable transcription of *pri-MIR828* would be one of the primary mechanisms. However, accumulation profiles of mature miR828 and *pri-MIR828* transcripts were different: the amounts of mature miR828 increased during flower bud growth, whereas those of *pre-MIR828* decreased with flower bud development. Therefore, processing and stability of microRNA were also potentially involved in the predominant accumulation of mature miR828 in the lower halves, and the post-transcriptional regulation of miR828 accumulation unlikely occurs in the upper halves because the amounts do not increase with bud development. MicroRNA abundance is regulated under multiple levels of control, including processing, RISC assembly, and stability (Wang et al., 2019). Different AGO proteins have different effects on the stability of their bound microRNAs, and spatially and temporally distinct AGO5 expression is indispensable for organ-specific function of miR156 (Roussin-Léveillée et al., 2020).

MYB12 has been found to be the primary transcription factor regulating bicoloration (Suzuki et al., 2016), and we experimentally confirmed the predominant cleavage of *MYB12* mRNA in the lower halves of tepals. However, compared with the upper halves, the levels of accumulated *MYB12* transcript in tepals were observed to be reduced by 40% in the lower halves, in which the expression of biosynthesis genes was markedly suppressed (Figure 4; Suzuki et al., 2016). We suspect that the observed differences in suppression levels could be attributable to the fact that miR828 not only cleaves the transcripts but also arrests *MYB12* translation in lily cells. In addition to the cleavage of target mRNA, translational attenuation of target genes is involved in microRNA-mediated gene regulation (Dalmay, 2013), and it is often reported that suppression of the accumulation of target gene transcripts is weaker than that of downstream genes regulated by the target genes (Gandikota et al., 2007; Wang et al., 2016; Gattolin et al., 2018).

Sequences of mature miR828 are highly conserved in many plants, including lilies, with an exception in *Arabidopsis*, in which one nucleotide is different. Major targets of miR828 are R2R3-MYB transcription factor genes and *TAS4*: *MdWEREWOLF* (*WER*, subgroup 15 member of R2R3-MYB), *MdC1* (R2R3-MYB), and *MdTAS4* in apple (Xia et al., 2012), *AtMYB113* (subgroup 6 member of R2R3-MYB), *AtMYB82* (ungrouped R2R3-MYB), and *AtTAS4* in *Arabidopsis* (Rajagopalan et al., 2006), *VvMYB114* (WER like MYB having EAR suppressor motif) and *VvWER* in *Vitis vinifera* (Tirumalai et al., 2019), *PpMYB* (subgroup 15 member of R2R3-MYB), two *PpMYB* (ungrouped MYB), and *PpTAS4* in peach (Zhu et al., 2012), and *R2R3-MYB* and *StTAS4* in potato (Bonar et al., 2018). R3 repeat sequences in *R2R3-MYB* genes are highly conserved, and the target site of miR828 exists near the end of the R3 repeat. Therefore, miR828 often targets several *MYB* genes (Xia et al., 2012; Zhu et al., 2012; Li and Lu, 2014). In Lollypop, 15 sequences were predicted to be targets, although whether they were cleaved by miR828 has not yet been evaluated, and five of the 15 sequences were *R2R3-MYB* (Supplementary Table S2). *MYB15*-like exhibited homology with *LrMYB15* in *Lilium regale*, which regulates bud blush anthocyanin pigmentation (Yamagishi, 2016), while, in Asiatic hybrid lilies, its expression levels are not high and its function is unknown (Yamagishi, 2018). *MYB16* is also expressed weakly in tepals, and its function is unknown (Yamagishi, 2018). Zm1-like Myb-related protein is a subgroup 2 member of *R2R3-MYB* (Suzuki et al., 2016). Although its function in lily is unknown, subgroup 2 members are not involved in pigmentation in other species. *MYB19S* is predominantly expressed in stage 2 flower buds and is involved in the pigmentation at raised spots developed at the basal parts of tepals (Yamagishi, 2020b). Therefore, the *R2R3-MYB* genes, other than *MYB12*, are unlikely to be involved in bicoloration.

The involvement of miR828 in pigment accumulation has been reported. MiR828 highly accumulates in pigmented grape fruits and potato tubers and inhibits the expression of grape *MYB114* and potato *R2R3-MYB*, which act as suppressors of anthocyanin biosynthesis (Bonar et al., 2018; Tirumalai et al., 2019). In lilies, miR828 accumulates in the unpigmented regions of tepals and suppresses the positive regulation of anthocyanin biosynthesis. Therefore, the phenomenon in lilies is distinct from those observed in grapes and potatoes.

Subgroup 6 members of R2R3-MYB in numerous species and subgroup 5 members of that in Poaceae and orchids are major positive regulators of anthocyanin biosynthesis. To determine whether miR828 targets these positive regulator genes, putative miR828 target sites found in the genes were compared (Supplementary Figure S4). Base-pairing between miR828 and the putative target sites showed that the number of mismatched nucleotides was three or fewer in *R2R3-MYB* genes of oil palm, onion, *Arabidopsis*, snapdragon, and *Salvia*. In *Arabidopsis*, *AtMYB113* is the direct target of miR828 (Luo et al., 2012), and *AtPAP1* and *AtMYB113* expression is suppressed in miR828-overexpressed *Arabidopsis* plants (Yang et al., 2013), indicating that miR828 regulates anthocyanin accumulation in *Arabidopsis* through the suppression of the positive regulators. However, as miR828 predominantly accumulates in siliques (Rajagopalan et al., 2006), miR828 is unlikely to be involved in flower coloration in *Arabidopsis*. Deep sequencing analysis revealed that *SmMYB36*, a subgroup 6 *R2R3-MYB* in *Salvia*, is a target of miR828 (Li and Lu, 2014), although the function of *SmMYB36* has not been clarified yet. Numerous mismatched nucleotides have been observed in the sequences of other eudicot species, including petunia, grape, and apple, suggesting that miR828 does not target such positive regulators directly in these species. Consequently, the bicolor formation mediated by the modules of miR828 and subgroup 6 members of R2R3-MYB, observed in lily flowers, should be rare in eudicots. Conversely, in addition to the lily *MYB*s, two of the three subgroup 6 members of *R2R3-MYB* (*EgVIR* in oil palm and *AcMYB1* in onion) in monocots are putative targets of miR828, although the number of subgroup 6 members of *R2R3-MYB* genes isolated and characterized in monocots is limited. Therefore, further investigation of miR828-mediated pattern formation should provide novel insights in monocots. Subgroup 5 members of the *R2R3-MYB*s are unlikely targets of miR828.

Several mechanisms of the development of anthocyanin color patterns have been clarified in flowers. The major mechanisms are spatially distinct expression of R2R3-MYB positive regulators in single petals (Fattorini and Glover, 2020), post-transcriptional gene silencing of *CHS* (Morita et al., 2012), and the insertion of transposable elements into anthocyanin biosynthesis genes (Momose et al., 2013; Uchiyama et al., 2013). MicroRNA-mediated pattern formation, presented in this study, is likely the novel mechanism of development of the attractive color patterns.

## Data Availability Statement

These deep sequencing files have been deposited in the DDBJ Sequence Read Archive under the accession number DRA010576.

## Author Contributions

MY designed the research, conducted the experiments, and wrote the manuscript. MS and MY analyzed the data. Both the authors have read and approved the submission of the manuscript.

### Conflict of Interest

The authors declare that the research was conducted in the absence of any commercial or financial relationships that could be construed as a potential conflict of interest.

## Footnotes

^1^https://www.genome.jp/tools-bin/clustalw

^2^https://www.R-project.org/

^3^https://expressionanalysis.github.io/ea-utils/

^4^https://rna.urmc.rochester.edu/RNAstructureWeb/Servers/Predict1/Predict1.html

^5^http://plantgrn.noble.org/psRNATarget/
