# Supplementary material for: The MicroRNA828/MYB12 Module Mediates Bicolor Pattern Development in Asiatic Hybrid Lily (Lilium spp.) Flowers
Source: Front Plant Sci. 2020 Oct 30;11:590791. doi: 10.3389/fpls.2020.590791 (PMC7661471; doi:10.3389/fpls.2020.590791)
Supplement: Supplementary file 1 [file Data_Sheet_1.pdf]

**Supplementary Table S1** PCR primers and an RNA adapter (5′ to 3′)

| | | |
|---|---|---|
| RACE PCR | | |
| poly(T)-adaptor primer | GGCGACCCCTCGACTAGATGCGGCCGCTTTTTTTTTTTTTTTTT | |
| 3′RACE, 1st PCR | TTCCTCCCACATGGGAAAGC | GGCGACCCCTCGACTAGATG |
| 3′RACE, 2nd (nested) PCR | TCCATCTATGGTGCAGAAGC | TCGACTAGATGCGGCCGC |
| 5′RACE, 1st PCR | GGCGACCCCTCGACTAGATG | CTCTGCTCATACACAACATCAC |
| 5′RACE, 2nd (nested) PCR | TCGACTAGATGCGGCCGC | TGTCTCGATATGGAGCTGGA |
| 5′RACE, 3rd (nested) PCR | TCGACTAGATGCGGCCGC | TCAGGCAAGCATAGGTTACC |
| RLM-RACE PCR | | |
| RNA adapter | GCUGAUGGCGAUGAAUGAACACUGCGUUUGCUGGAUGAAA | |
| reverse transcription | TGTTTACATGGACCTTGCTAT | |
| 1st PCR | GCTGATGGCGATGAATGAACACTG | TATCAGAAAGCAAGCCGTCC |
| 2nd (nested) PCR | TGAACACTGCGTTTGCTGGATG | CGAATCCATTTGGTCATTCAT |
| 35S-p::pre-MIR828 construct (red letters indicate restriction sites) | | |
| | GACTCTAGATTTTCCTCCCACATGGGAAAG | TTCGAGCTCTCTTCTTCCTCCCACAGAATG |
| stem-loop pulsed PCR | | |
| reverse transcription | GTTGGCTCTGGTGCAGGGTCCGAGGTATTCGCACCAGAGCCAACTGGAAT | |
| end-point PCR | TCGCG*TCTTGCTCAAATGAGT* | GTGCAGGGTCCGAGGT |
| *U6* | CGGGGACATCCGATAAAATTGGAACG (U6af) | CGATTTGTGCGTGTCATCCTTGC (U6br) |
| qRT-PCR in tobacco (agroinfiltration) | | |
| *MYB12* | CTCTTGGGAAACAGGTGGTC | CAGCTTCACCCTGTTTCTTCATCCTCAACC |
| *NtDFR* | TGAGTTTAAAGGCATCGATAAGGA | GAATTGAAACCCCATATCCGTC |
| *iGUS* | TGGATCCCTACAGGTTACAG | GAATATCTGCATCGGCGAAC |
| *NtUBQ* | AAGATTCAGGACAAGGAAGGCA | AGCTGCTTACCTGCGAAAATCA |
| *Rluc* | ATCATGGGATGAATGGCCTG | GCAACATGGTTTCCACGAAG |
| qRT-PCR in lilies | | |
| *Pri-MIR828* | CCTCCAGCTCCATATCGAGAC | CTCTGCTCATACACAACATCAC |
| *MYB12* | GGGTGAAGCTGAACCAAAAA | CGAATCCATTTGGTCATTCAT |
| *CHSa* | GCGAAGCTGGGACTGCAGAAGG | CAAGACCACCGTTTCCACGGTT |
| *CHSb* | CTGAAGCTGGCGCTGGACAAAAAG | GGTAGTGATCGGAATGCTGTGAAGA |
| *F3H* | GGTGCCTTTGTCGTCAATCT | AACTTCGGTGGCTTCTTCG |
| *DFR* | AATGGTTGCACCGGTGTGTT | GCACGTTCACAGTTCCAGCA |
| *ANS* | GGTGGTGACCAAGATGCTGT | CCAATGTGGACGAGAAGGGA |
| *ACTIN* | GGAGTGAGCCACACAGTTCC | ATAGCTCTTCTCCACAGAGG |
| *MYB12* mRNA-cleavage products (Figure 5) | | |
| | GTTTGCTGGATGAAATTGAGC | CGAATCCATTTGGTCATTCAT |

**Supplementary Table S2** Putative targets of miR828 estimated using psRNATarget: A Plant Small RNA Target Analysis Server[1]. R2R3-MYB genes are shown in red.

| Accession #[2] | Target annotation | Blast top hit | Expectation[3] | UPE[4] | Target aligned fragment | Inhibition |
|---|---|---|---|---|---|---|
| c24386_g1_i2 | MYB15like (subgroup 6 R2R3-MYB) | LC218141.1 [Lilium hybrid division I (Asiatic hybrid lilies)] | 2 | 9.996 | UGGAACUCUCAUUUGAGCAAGA | Cleavage |
| c22900_g1_i4 | MYB12 (subgroup 6 R2R3-MYB) | AB534586.1 [Lilium hybrid division I (Asiatic hybrid lilies)] | 2.5 | 11.116 | UGGAAUUCUCACUUGAGCAAGA | Translation |
| c20211_g1_i1 | calcineurin-binding protein 1-like | XP_017701940.1 [Phoenix dactylifera] | 2.5 | 21.912 | CACGAUGGUCAUUUGAGCAAGG | Cleavage |
| c24386_g2_i1 | MYB16 (subgroup 6 R2R3-MYB) | LC218139.1 [Lilium hybrid division I (Asiatic hybrid lilies)] | 3 | 17.361 | UGGAACGCUCACUUGAGCAAGA | Translation |
| c28082_g1_i1 | ADP-ribosylation factor GTPase-activating protein | XP_010926864.1 [Elaeis guineensis] | 3 | 17.961 | CAAGAUCUUCAUUUGAGCAAGC | Cleavage |
| c29023_g1_i1 | ATP-dependent DNA helicase homolog RECG, chloroplastic | XP_010936813.1 [Elaeis guineensis] | 3.5 | 18.032 | UCGAACAUUUAGUUGAGCAAGA | Translation |
| c28053_g1_i1 | putrescine-binding periplasmic protein-like protein | NP_174426.2 [Arabidopsis thaliana] | 4 | 14.292 | UGGAAGGCUUAUUUGUGCAGGA | Cleavage |
| c12438_g1_i3 | myb-related protein Zm1-like (subgroup 2 R2R3-MYB) | XP_020251095.1 [Asparagus officinalis] | 4 | 20.706 | UGGAAUACCCAUUUGAAGAAGA | Cleavage |
| | MYB19S (subgroup 6 R2R3-MYB)[5] | LC519097 [Lilium hybrid division I (Asiatic hybrid lilies)] | 4 | 9.863 | UGGAAUUCACACUUGAGUAAGA | Translation |
| c30057_g1_i5 | cullin-associated NEDD8-dissociated protein-like | XP_008795267.1 [Phoenix dactylifera] | 4 | 14.624 | CUGAAUGCACAUUUGGGGGAGA | Cleavage |
| c27842_g1_i1 | nodulin homeobox-like | XP_008805054.1 [Phoenix dactylifera] | 4 | 15.802 | CCGAAAGUUCAUUUGGCCAAGA | Cleavage |
| c28282_g1_i1 | hypothetical protein | RWR81552.1 [Cinnamomum micranthum f. kanehirae] | 4 | 20.989 | CCUUAUGCUCAUGUGAGCAAGC | Translation |
| c30149_g1_i1 | E3 ubiquitin ligase PQT3-like | XP_010917745.1 [Elaeis guineensis] | 4 | 14.679 | UGCAAGAUCCAUUUGAGCAACA | Cleavage |
| c23173_g1_i1 | fumarylacetoacetase | XP_008776647.1 [Phoenix dactylifera] | 4 | 17.310 | GACAAUGCUAAUUUGAGGGAGA | Cleavage |
| c29559_g1_i1 | uncharacterized protein | XP_010912273.1 [Elaeis guineensis] | 4 | 23.804 | UAUAAUAUUCAUAUGAGGAGGA | Translation |

[1] http://plantgrn.noble.org/psRNATarget/
[2] Lollypop tepal transcriptome (Suzuki et al. 2016)
[3] Expectations lower than 4 are shown
[4] UPE: Target accessibility-maximum energy to unpair the target site (Less energy means more possibility that microRNA is able to cleave target mRNA)
[5] Yamagishi, 2020b

**Supplementary Table S3** Overview of the small RNA-seq

Triming summary

| Sample Name | # Reads | # Reads Pairs | # Excluded Read Pairs | Matched | | Mismatched | |
|---|---|---|---|---|---|---|---|
| | | | | # Pairs | % Pairs | # Pairs | % Pairs |
| Lower half | 43,917,562 | 21,958,781 | 2,454,028 | 18,887,232 | 86.01 | 617,521 | 2.81 |
| Upper half | 43,401,220 | 21,700,610 | 2,066,021 | 18,997,636 | 87.54 | 636,953 | 2.94 |

Mapping summary

| Sample Name | # Input Reads | # Total Mapped Reads | % of Total Mapped Reads |
|---|---|---|---|
| Lower half | 18,887,232 | 874 | 0.0046 |
| Upper half | 18,997,636 | 75 | 0.0004 |

```
                                                                          miR828
pri-MIR828-1   GAGCATCATCTCCATAATTTTCCTCCCACATGGGAAAGCCTCTTGCTCAAATGAGTATTCCATCTATG
pri-MIR828-2   GAGCATCATCTCCATAATTTTCCTCCCACATGGGAAAGCCTCTTGCTCAAATGAGTATTCCATCTATG

                                                                              miR828*
pri-MIR828-1   GTGCAGAAGCTTTGGGTCCTCTCGGAGCTTCATGCATTGCAGAAGTGGATGCTCATTAGGGTAAGATG
pri-MIR828-2   GTGCAGAAGCTTTGGGTCCTCTCGGAGCTTCATGCATTGCAGAAGTGGATGCTCATTAGGGTAAGATG

pri-MIR828-1   CATTCTGTGGGAGGAAGAAGAGATATTCAAATGGATGGCCAACAGGTTCTCAGCAGCATAACTCTCTG
pri-MIR828-2   CATTCTGTGGGAGGAAGAAGAGATATTCAAATGGGTGGCCAACAGGTTCTCAGCAGCATAACTCTCTG

pri-MIR828-1   ATTTCTCCGCTTATTTTTGGTAGCCTATGCTTGCCTGAGTGCCCCTCTGGTACGAGTGAGCCCCCATC
pri-MIR828-2   ATTTCTCCTCT-ATCTTTGGTAACCTATGCTTGCCTGAGTGCCCCTCTGGTACGAGTGAGCTCCCATC

pri-MIR828-1   CCCTCCATGCAAAATCCCTCCCCGACTTATCATATACCTTCATCTTGTTAATTGTTTTCTGTGTCTCC
pri-MIR828-2   CCCTCCATGCAAAATCCCTCCCCGACTTATCATATACCTTCATCTTGTTAATTGTTTTCTGTGTCTCC

pri-MIR828-1   TCCTCCAGCTCCATATCGAGACAATCACACAGCCCACACCTGCGCCGACACTGAGGCAAGCAGTGCCG
pri-MIR828-2   TCCTCCAGCTCCATATCGAGACAATCACACAGCCCCCACCTGCGCCGACACTGAGGCAAGCAGTGCCG

pri-MIR828-1   CCCTCCAGCTACTCAGCATCTCCTTAAACCTTCAAAGTTTCTGGTGTTGTGCATGGGCCCGAGAGTCG
pri-MIR828-2   CCCTCCAGCTACTCAGCATCTCCTTAAACCTTCAAAGTTTCTGGTGTTGTGCTTGGGCCCGAGAGTCG

pri-MIR828-1   GCAATTGCACCTAAACCTGCAAAGTACCTGTGATGTTGTGTATGAGCAGAGGGGCGGGTATCTACACC
pri-MIR828-2   GCAATTGCACCTAAACCTGCAAAGTACCTGTGATGTTGTGTATGAGCAGAGGGGCGGGTACCTACACC

pri-MIR828-1   TCCAAAAAGTCTATGGCCAGGGGGCGGCAACTGTACCTACATCTAAAAAAAATTAAATTAAATTAAAT
pri-MIR828-2   TCCAAAAAGTCTATGGCCAGGGGGCGGCAACTGTACCTACACCTAAAAAAGATTAAATTAAATTAAAT

pri-MIR828-1   TAAAAAGACCTTCTGGTTCTTAGT--------------------------------------------
pri-MIR828-2   TAAAAAGACCTTCTGGTGCTTAGTATGTTAGAATTAATCTTGTTATAATTCTTTTAAATAAAACGTGG

pri-MIR828-1   -----------------AAAAAAAAAAAAAAAAAA
pri-MIR828-2   TTACTGTCTTGAATACCAAAAAAAAAAAAAAAAAA
```

**Supplementary Figure S1** Nucleotide sequence alignment of *pri-MIR828* in Lollypop. Guide (miR828) and passenger (miR828*) strand sequences are shown in red background.

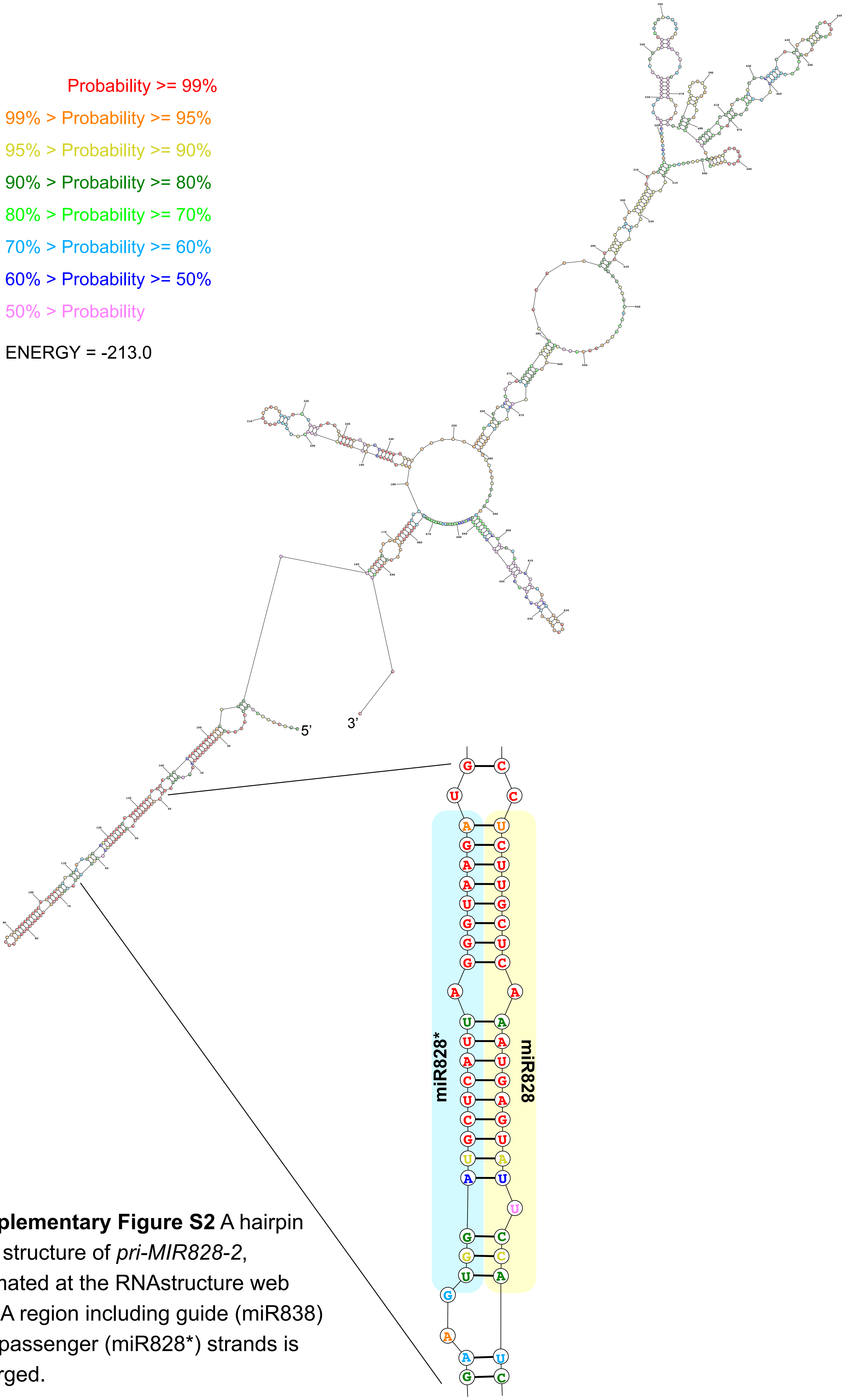


**Supplementary Figure S2** A hairpin loop structure of *pri-MIR828-2*, estimated at the RNAstructure web site. A region including guide (miR838) and passenger (miR828*) strands is enlarged.

A

```
TGAACACTGCGTTTGCTGGATGAAATTGAGCAAGAGAAAAGTGAATGTCGAACA···
TGAACACTGCGTTTGCTGGATGAAATTGAGCAAGAGAAAAGTGAATGTCGAACA···
TGAACACTGCGTTTGCTGGATGAAATTGAGCAAGAGAAAAGTGAATGTCGAACA···
TGAACACTGCGTTTGCTGGATGAAATTGAGCAAGAGAAAAGTGAATGTCGAACA···
TGAACACTGCGTTTGCTGGATGAAATTGAGCAAGAGAAAAGTGAATGTCGAACA···
TGAACACTGCGTTTGCTGGATGAAATTGAGCAAGAGAAAAGTGAATGTCGAACA···
TGAACACTGCGTTTGCTGGATGAAATTGAGCAAGAGAAAAGTGAATGTCGAACA···
TGAACACTGCGTTTGCTGGATGAAATTGAGCAAGAGAAAAGTGAATGTCGAACA···
TGAACACTGCGTTTGCTGGATGAAATTGAGCAAGAGAAAAGTGAATGTCGAACA···
```

B

```
TGAACACTGCGTTTGCTGGATGAAA-----TTGAGCAAGAGAAAAGTGAATGTCGAACA···
TGAACACTGCGTTTGCTGGATGAAA-----TTGAGCAAGAGAAAAGTGAATGTCGAACA···
TGAACACTGCGTTTGCTGGATGAAA-----TTGAGCAAGAGAAAAGTGAATGTCGAACA···
TGAACACTGCGTTTGCTGGATGAAA--------AGCAAGAGAAAAGTGAATGTCGAACA···
TGAACACTGCGTTTGCTGGATGAAA-----TTGAGCAAGAGAAAAGTGAATGTCGAACA···
TGAACACTGCGTTTGCTGGATGAAACTCACTTGAGCAAGAGAAAAGTGAATGTCGAACA···
TGAACACTGCGTTTGCTGGATGAAA-----TTGGGCAAGAGAAAAGTGAATGTCGAACA···
TGAACACTGCGTTTGCTGGATGAAA--------AGCAAGAGAAAAGTGAATGTCGAACA···
```

C

```
TGAACACTGCGTTTGCTGGATGAAATTGAGCAAGAGAAAAGTGAATGTCGAACA···
TGAACACTGCGTTTGCTGGATGAAATTGAGCAAGAGAAAAGTGAATGTCGAACA···
TGAACACTGCGTTTGCTGGATGAAATTGAGCAAGAGAAAAGTGAATGTCGAACA···
TGAACACTGCGTTTGCTGGATGAAATTGAGCAAGAGAAAAGTGAATGTCGAACA···
TGAACACTGCGTTTGCTGGATGAAATTGAGCAAGAGAAAAGTGAATGTCGAACA···
TGAACACTGCGTTTGCTGGATGAAATTGAGCAAGAGAAAAGTGAATGTCGAACA···
TGAACACTGCGTTTGCTGGATGAAA---AGCAAGAGAAAAGTGAATGTCGAACA···
TGAACACTGCGTTTGCTGGATGAAATTGAGCAAGAGAAAAGTGAATGTCGAACA···
TGAACACTGCGTTTGCTGGATGAAATTGAGCAAGAGAAAAGTGAATGTCGAACA···
TGAACACTGCGTTTGCTGGATGAAATTGAGCAAGAGAAAAGTGAATGTCGAACA···
```

**Supplementary Figure S3** Sequences of the RLM-RACE PCR products of *MYB12*. Nucleotides corresponding to the RNA adapter are shown in blue letters and those corresponding to *MYB12* sequences are shown in black letters. A: RNA was isolated from tobacco leaves infiltrated with the 35S-p::*MIR828* and 35S-p::*MYB12* constructs. B: RNA was derived from tobacco leaves infiltrated with the 35S-p::*MIR828*, 35S-p::*MYB12* and other constructs. C: RNA was isolated from the lower halves of Lollypop tepals at stage 4. The results shown in A, B, and C are summarized in Figure 2B, 2D, and 3B, respectively.

| | | | microRNA828 in Arabidopsis: 3'- ACCTTATGAGTAAATTCGTTCT -5'<br>microRNA828 in other plants: 3'- ACCTTATGAGTAAACTCGTTCT -5' | | Expectation | UPE | Inhibition |
|---|---|---|---|---|---|---|---|
| Monocots | Lily | MYB12 | 5'- ATATTAAGAACTACTGGAATTCTCACTTGAGCAAGAGAAAAGTGAATGTC -3' | (2) | 2.5 | 11.1 | Translation |
| | | MYB19S | 5'- ATATAAAAAACTACTGGAATTCACACTTGAGTAAGAAAAAAGTGAATGTT -3' | (3) | 4 | 9.9 | Translation |
| | | MYB15like | 5'- ACGTAAAGAACCACTGGAACTCTCATTTGAGCAAGAAACTCATTTCCGGA -3' | (2) | 2 | 10.0 | Cleavage |
| | | MYB16 | 5'- ACGTGAAGAACTACTGGAACGCTCACTTGAGCAAGAAAGTTGTAGCTCGA -3' | (2) | 3 | 17.4 | Translation |
| | *Anthurium* | AaMYB2 | 5'- ACATCAAGAACTATTGGAACTCGTACTTGGGCAAGAAGGTAGTTTCCCAG -3' | (5) | | | |
| | Oil palm | EgVIR | 5'- ACATCAAGAACTACTGGAACACTCACCTGGGCAAGAAAGTAG-------- -3' | (3) | 4.5 | 13.2 | Translation |
| | Onion | AcMYB1 | 5'- ACATAAAAAACTTCTGGAACACTCACCTGAGCAAGAAGAAGT------TA -3' | (3) | 4 | 13.9 | Translation |
| | *Phalaenopsis* | PeMYB2 | 5'- AAATAAAGAACTACTGGAACACAACCTTGGCGAAGAAGACAACCTCAC-- -3' | (7) | | | |
| | | PeMYB12 | 5'- AGATAAAAAATTACTGGAACACAACCTTAGGTAAAAAGCTTCGC----AA -3' | (7) | | | |
| | | PeMYB11 | 5'- AGATCAAGAACTATTGGAACACGACTCTATCGAAGAAGATTCAAACAA-- -3' | (9) | | | |
| Eudicots | *Arabidopsis* | AtPAP1 | 5'- ACGTCAAGAATTACTGGAACACTCATCTGAGTAAGAAACA---TGAACC- -3' | (2) | 3 | 16.7 | Translation |
| | | AtPAP2 | 5'- ATGTCAAAAATTACTGGAACACCCATCTGAGTAAAAAACA---TGAGTCT -3' | (4) | | | |
| | | AtMYB113 | 5'- ATGTCAAGAATTACTGGAACACTCATTTGAGTAAGAAGCA---CGATGAA -3' | (1) | 1.5 | 17.5 | Cleavage |
| | | AtMYB114 | 5'- ATGTCAAGAACTACTGGAACACCCATCTGAGTAAGAAGCA---TGAACC- -3' | (3) | 4 | 16.1 | Translation |
| | Petunia | PhAN4 | 5'- ATGTGAAAAACTACTGGAACACTAACCTTCTAAGGAGGTCAAAGTTTGCT -3' | (8) | | | |
| | | PhDPL | 5'- ATGTGAAAAACTACTGGAATACTCAACTTCTAAGGAGGTCAAACTTTGCT -3' | (6) | | | |
| | | PhPHZ | 5'- ATGTCAAAAATTACTGGAACACACACCTGCAGAGGAAGTTAA---TTGCT -3' | (7) | | | |
| | | PhAN2 | 5'- ATGTCAAAAACTATTGGAACACCCACCTTCGAAAGAAGTTAATTGCTCCT -3' | (7) | | | |
| | Snapdragon | AmROSEA1 | 5'- ACGTGAAGAACTTTTGGAATACTCATGTGGGGAAGAATTTAG------GC -3' | (2) | 3.5 | 14.4 | Translation |
| | Apple | MdMYB10a | 5'- CTGTGAAAAATTATTGGAACACTCGATTGCGGATCGATTC---------- -3' | (6) | | | |
| | Grape | VlMYBA1-1 | 5'- ATGTCAAGAACTATTGGCATGGTCACCATTTGAAAAAGAAGG------TT -3' | (10) | | | |
| | | VlMYBB1-1 | 5'- AGATAAAAAATGTCTGGCACACACACCTGAAGAAGAGGCTCAAGAAAAAA -3' | (7) | | | |
| | *Salvia* | SmMYB36 | AGATCAAGAACTACTGGAATGCTCACTTGAGGAAGAAAGC---CATGTTG -3' | (2) | 3.5 | 14.1 | Translation |

R3 repeat

**Supplementary Figure S4** Putative miR828 target sites in subgroup 5 members (*Phalaenopsis*) and subgroup 6 members (other species) of R2R3-MYB sequences regulating anthocyanin biosynthesis in monocots and eudicots. Number of mismatched nucleotides is shown in parentheses. Expectation, UPE, and Inhibition were estimated using "psRNATarget: A Plant Small RNA Target Analysis Server" (see Supplementary Table S2). Expectations lower than 5 are shown. The R3 repeat region is represented by a blue bar.

Accession numbers (in parentheses) are *AaMYB2* (KU726561) in *Anthurium andraeanum*, *Virescens* (EgVIR, KJ789862) in *Elaeis guineensis*, *AcMYB1* (KX785130) in *Allium cepa*, *PeMYB12* (AIS35929), *PeMYB2* (AIS35919), and *PeMYB11* (AIS35928) in *Phalaenopsis equestris*, *AtPAP1* (NM_104541), *AtPAP2* (NM_105310), *AtMYB113* (NM_105308), and *AtMYB114* (NM_105309) in *Arabidopsis thaliana*, *PhAN2* (AF146702), *PhAN4* (HQ428105), *PhDPL* (HQ428109), and *PhPHZ* (HQ428103) in *Petunia hybrida*, *AmROSEA1* (DQ275529) in *Antirrhinum majus*, *MdMYB10a* (DQ267896) in *Malus × domestica*, *VlMYBA1-1* (AB073010) and *VlMYBB1-1* (AB073016) in *Vitis labrusca × V. vinifera*, and *SmMYB36* (KF059390) in *Salvia miltiorrhiza*.
